# Sulfated Polysaccharides From *Gracilaria gracilis* (Red Seaweed) Restores Testicular Glucose Metabolism by Improving FSH and Insulin Signaling in Rats With Type 2 Diabetes

**DOI:** 10.1002/mnfr.70280

**Published:** 2025-10-05

**Authors:** Ochuko L. Erukainure, Tosin A. Olasehinde, Olanrewaju S. Olayeriju, Bukola Omotoso, Md. Shahidul Islam, Ademola O. Olaniran

**Affiliations:** ^1^ Department of Microbiology School of Life Sciences University of KwaZulu‐Natal Durban South Africa; ^2^ Department of Biochemistry School of Life Sciences University of Kwazulu‐Natal Durban South Africa; ^3^ Nutrition and Toxicology Division Food Technology Department Federal Institute of Industrial Research Oshodi Lagos Nigeria; ^4^ Chemical Science Department, Biochemistry Unit Ondo State University of Science and Technology Okitipupa Ondo Nigeria; ^5^ Department of Internal Medicine Faculty of Health Sciences University of the Free State Bloemfontein South Africa

**Keywords:** FSH signaling, glycolytic‐gluconeogenic switch, *Gracilaria gracilis*, insulin signaling, sulfated polysaccharides

## Abstract

Testicular glucose homeostasis plays important roles in testicular energy metabolism and male fertility, but it is altered in type 2 diabetes (T2D), leading to male infertility. In the present study, the therapeutic effect of sulfated polysaccharides (SPCs) from *Gracilaria gracilis* on testicular glucose metabolism was investigated in T2D rats. SPCs were administered to two groups of T2D rats at 150 and 300 mg/kg bodyweight, respectively. The T2D control group was administered water only, while metformin served as the control drug administered to the standard treatment group. Non‐T2D rats served as the normal group. After 5 weeks of treatment, the rats were sacrificed and their testes harvested and analyzed for insulin, FSH, glucose, and glutathione metabolisms. Treatment with SPCs led to a significant increase in insulin, IRS‐1, FSH, GLUT4, GSH, ATP levels, hexokinase, glucose 6‐phisphate dehydrogenase, glyoxalase, glutathione reductase, and glutathione peroxidase activities, while improving testicular morphology. SPCs further led to significant depletion in glycogen phosphorylase, glucose 6‐phosphatase, fructose‐1,6‐biphosphatase, aldose reductase, polyol dehydrogenase, ATPase, and ENTPDase activities. These results indicate the potentials of SPCs from *G. gracilis* to improve male fertility in T2D. This is evident by its ability to improve insulin‐FSH signaling, glucose, energy metabolisms, and testicular morphology.

## Graphical abstract

Sulfated polysaccharides from red seaweed improved testicular FSH and insulin signaling, which upregulated GLUT4 level and improved glycolysis and energy metabolism in testes of diabetic rats.

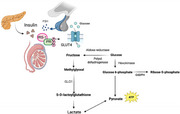

## 1 Introduction

Testicular dysfunction leading to male infertility is among the complications associated with type 2 diabetes (T2D). This has been attributed to distortion in testicular glucose homeostasis, which affects spermatogenesis [1]. This is characterized by impaired ATP production via anerobic glycolysis, required for capacitation, fertilization capability, and sperm motility [2, 3]. Downregulation of testicular glucose transporters leading to impaired glucose uptake has also been implicated in glucose dysmetabolism in testes [4]. These glucose transporters are sensitive to insulin [5, 6]. Insulin has also been shown to have a direct effect on gonadal cells [7]. Plasma insulin crosses the blood‐testis barrier (BTB) and directly influences steroidogenesis in Leydig cells [8]. Insulin receptors (insulin receptor [INSR] and insulin‐like growth factor 1 receptor [IGF1R]), insulin receptor substrate 1 (IRS‐1), and insulin receptor substrate 2 (IRS‐2) are expressed in testicular endothelial cells, peritubular myoid cells, Sertoli cells, and spermatocytes [9, 10]. Insulin plays a crucial role in testicular development, including spermatogenesis, libido, and proliferation and differentiation of Sertoli cells [10, 11, 12]. The follicle‐stimulating hormone (FSH), secreted by the pituitary gland, also plays multifaceted roles in male fertility. FSH stimulates spermatogenesis, translocation of glucose transporter 4 (GLUT4), and activation of hexokinase activity [10, 13, 14]. Both insulin and FSH exert their action via phosphorylation of IRS‐1, which triggers downstream pathways that drive the expression of genes involved in follicle maturation and spermatogenesis [15]. Down regulation of IRS‐1 in testicular tissues, leading to alteration in FSH and insulin signaling, has been implicated in impaired testicular functions in T2D [15, 16].

Over the years, there has been an increasing interest in the use of natural products in the treatment of various diseases, including T2D and reproductive dysfunctions [17, 18]. This paradigm shift has been attributed to several factors, including their little or no side effects as well as their accessibility, affordability, and availability [19, 20, 21]. Amongst these natural products are sulfated polysaccharides (SPCs), with seaweeds being their major sources. Sulfated polysaccharides are complex carbohydrates with attached sulfate groups and have been reported for their medicinal properties [22, 23]. Sulfated polysaccharides have been reported for their antidiabetic properties, which have been attributed to their ability to inhibit the activities of carbohydrate digestive enzymes, stimulate muscle glucose uptake, improve oral glucose tolerance, maintain insulin secretion and sensitivity, suppress fasting blood glucose level, and protect pancreatic‐β cells [23, 24, 25, 26, 27, 28]. The use of SPCs in the management of male reproductive dysfunction has been attributed to their ability to mitigate testicular oxidative stress and autophagy, maintain testicular morphology, improve production and secretion of LH and FSH, and improve sperm quality [29, 30, 31].

*Gracilaria gracilis* is among the red seaweed species in the *Gracilaria* genus and a rich source of SPCs. Native to South Africa, there is limited scientific evidence of *G. gracilis* medicinal properties. In our previous studies, we demonstrated the ability of SPCs from *G. gracilis* to scavenge free radicals, inhibit glucose‐metabolizing enzymes, and promote muscle glucose uptake in vitro [27]. FT‐IR analysis showed that the SPCs consisted of O–H, C–H, and S = O functional groups, with C–O–S and C–O–C stretches indicating the presence of d‐galactose and 3,6‐anhydrogalactose, respectively [27]. Aside from this study, there is a dearth of information on the effect of *G. gracilis* SPCs on male reproductive dysfunction in T2D. Thus, the present study was undertaken to investigate the effect of SPCs from *G. gracilis* on glucose metabolism, insulin signaling, and FSH secretion in testicular tissues of T2D rats.

## 2 Materials and Methods

### 2.1 Collection of *G. gracilis*

*G. gracilis* was collected from Wild Coast Abalone, East London, South Africa and was identified at the herbarium of the Department of Botany, Nelson Mandela University, South Africa (32°45.048′S. 28°16.558ʹE), where it was deposited and assigned the voucher code, D1.

The seaweed sample was rinsed off salt and sand particles with distilled water and air‐dried before lyophilizing in a freeze‐dryer. The freeze‐dried samples were blended into a fine powder, and 200 g was defatted by macerating in 1000 mL n‐hexane with constant shaking for 24 h. The solvent was decanted, and the residue was dried overnight in a fume cupboard [27].

### 2.2 Extraction of Sulfated Polysaccharides

The sequential cold‐ and hot‐water extractions and ethanol precipitation methods were utilized in extracting SPCs from the seaweed sample as described by Pengzhan et al. [32] and Olasehinde et al. [33]. For cold‐water extraction, 100 g of the defatted sample was extracted with 500 mL of distilled water overnight at room temperature. A Büchner funnel lined with a Whatman TG 100 Separating Gauze was used in filtering the slurry. The filtrate was mixed with 2 volumes of absolute ethanol in a 2 L Erlenmeyer flask and allowed to precipitate overnight at 4°C. The mixture was centrifuged at 4000 × *g* for 5 min. The precipitated pellets (SPCs) were collected and lyophilised by freeze‐drying. The lyophilized SPCs were blended into powder and stored at 4°C until further analysis.

For the hot‐water extraction, 100 g of the defatted seaweed sample was mixed with 500 mL of distilled water and autoclaved for 15 min. A Büchner funnel lined with a Whatman TG 100 Separating Gauze was used to filter the slurry. The resulting filtrate was subjected to SPCs extraction using the same protocol above.

### 2.3 Monosaccharide Composition

We have previously reported the monosaccharide composition of SPCs from *G. gracilis* following LC‐MS and NMR analyses [27]. Glucose, mannuronic acid, galactose, arabinose, xylose, and glucuronic acid were identified as the monosaccharide composition in the ratio of 185:141:21:12:1:142.

### 2.4 Animals

Thirty‐four (34) male Sprague Dawley (SD) rats weighing 180–200 g were obtained from the Biomedical Research Unit (BRU), University of KwaZulu‐Natal, Durban, South Africa. They were acclimatized on pelletized chows and water (ad libitum) for 7 days, and subjected to natural photo period of 12‐h light–dark cycle. The study was carried out under the approval of the Animal Ethics Committee, University of KwaZulu‐Natal, Durban, South Africa (Ethical Approval Number: AREC/00002347/2021).

### 2.5 Animal Groupings

After acclimatization, the animals were grouped into six, namely Normal Control (NC): normal rats (non‐diabetic); Diabetic Control (DC): untreated diabetic rats; Diabetic + low dose (DRL): diabetic rats administered low dose (150 mg/kg bodyweight [bw]) of SPCs; Diabetic + high dose (DRH): diabetic rats administered high dose (300 mg/kg bw) of SPCs; Diabetic + standard drug metformin (DBM): diabetic rats administered 200 mg/kg bw of metformin; and Normal Toxicological Control (NTR): non‐diabetic rats administered high dose of SPCs. The selected doses were based on results from preliminary in vitro and ex vivo studies from the lab.

The normal groups (NC and NTR) consisted of five rats each, while the others consisted of six rats each.

### 2.6 Induction of Type 2 Diabetes

Following adaptation, the diabetic groups were induced with T2D using the fructose‐streptozotocin model [34]. Briefly, 10% fructose was provided ad libitum to the diabetic groups (DC, DRL, DRH, and DBM) for 2 weeks. Following overnight fasting, the rats were injected intraperitoneally with streptozotocin (40 mg/kg bw) in citrate buffer (pH 4.5). The normal groups (NC and NTR) were intraperitoneally injected with citrate buffer only.

After 7 days, non‐fasting blood glucose levels were measured with a glucometer (Accu‐Chek), and rats with blood glucose level > 200 mg/dL were considered diabetic.

### 2.7 Intervention Trial

Following induction of T2D, DRL, DRH, and NTR groups were orally administered SPCs via oral gavage. Distilled water was to NC and DC groups, while DBM was administered metformin. Treatment was carried out five times a week for an intervention period of 5 weeks.

### 2.8 Sacrifice and Collection of Testes

Prior to sacrifice, the rats were placed in metabolic cages and fasted overnight (8 h). Their urines were collected into sterile tubes and stored at −80°C for further analysis. The rats were humanely sacrificed by euthanizing with Isoform. Blood was collected via cardiac puncture into sterile plain tubes, and the rats were dissected. Their testes were harvested and rinsed off blood stains with normal saline. About a 5 mm section was excised from each testis and fixed in 10% neutral buffered formalin for histological analysis. About 0.5 g of each testis was excised and homogenized in 5 mL of sodium phosphate buffer (pH 7.5; 50 mM) containing 10% Triton X‐100. The homogenized samples were centrifuged for 10 min at 15 000 rpm, 4°C. The supernatants were collected into 2 mL Eppendorf tubes and stored at −80°C for further analysis.

The collected blood was centrifuged at 15 000 rpm, 4°C for 10 min. The supernatants, which are serums, were collected into 2 mL Eppendorf tubes and stored at −80°C for further analysis.

### 2.9 Follicle‐Stimulating Hormone Levels

FSH levels were measured in the serum, testicular tissues, and urine using an FSH assay kit (Elabscience, Houston, TX, USA; Catalog No: E‐EL‐R0391) according to the manufacturer's instructions.

### 2.10 Testicular Insulin Receptor Substrate 1 (IRS‐1)

Testicular levels of IRS‐1 were measured using an IRS‐1 ELISA kit (Thermo Fisher Scientific, Waltham, MA, USA; Catalog No. KH00521) according to the manufacturer's instructions.

### 2.11 Testicular Insulin

Testicular insulin level was measured using an insulin ELISA kit (Merck, Johannesburg, South Africa; Catalog No: EZRMI‐13K) according to the manufacturer's instructions.

### 2.12 Glucose Transporter 4 (GLUT4) Level

Testicular levels of GLUT4 were measured using a GLUT4 ELISA kit (Elabscience, Houston, TX, USA; Catalog No: E‐EL‐R0430) according to the manufacturer's instructions.

### 2.13 Hexokinase Activity

Testicular hexokinase activity was determined using a hexokinase (HK) ELISA kit (Elabscience, Houston, TX, USA; Catalog No: E‐BC‐K610‐M) according to the manufacturer's instructions.

### 2.14 Glucogenic Enzyme Activities

The glucogenic enzyme activities, which cover fructose‐1,6‐biphosphatase, glucose 6‐phosphatase, and glycogen phosphorylase activities, were determined in the testicular tissues using previously described protocols [35, 36, 37, 38, 39].

#### 2.14.1 Fructose‐1,6‐Bisphosphatase Activity

Briefly, 100 µL of the tissue supernatant was incubated with 100 µL of 0.05 M fructose, 1200 µL of 0.1 M Tris–HCl buffer (pH 7.0), 250 µL 0.1 M MgCl_2_, 100 µL 0.1 M KCl, and 250 µL 1 mM EDTA at 37°C for 15 min. The reaction was stopped with 10% TCA and centrifuged at 3000 rpm (4°C) for 10 min. Fifty microliter of freshly prepared 9% ascorbic acid and 1.25% ammonium molybdate were mixed with 100 µL of the resulting supernatant in a 96‐well plate. The reaction mixture was allowed to stand for 20 min at room temperature, and absorbance was measured at 680 nm.

#### 2.14.2 Glucose 6 Phosphatase Activity

Briefly, 200 µL of the tissue supernatant was incubated with 100 µL of 0.25 M glucose 6‐phosphatase, 200 µL of 5 mM KCl, 1300 µL of 0.1 M Tris–HCl buffer in a shaker for 30 min at 37°C. The reaction was stopped with 1 mL of distilled water and 1.25% ammonium molybdate. One milliliter of freshly prepared 9% ascorbate was then added to the reaction mixture and allowed to stand for 30 min. Absorbance was measured at 660 nm.

#### 2.14.3 Glycogen Phosphorylase Activity

Briefly, 100 µL of the tissue supernatant was incubated with 64 mM glucose‐1‐phosphate and 4% glycogen and incubated for 10 min at 30°C. The reaction was stopped with 20% ammonium molybdate in concentrated H_2_SO_4_. The reaction mixture was further incubated with Elon reducer and distilled water at 30°C for 45 min. Absorbance was read at 340 nm.

### 2.15 Polyol Pathway

The polyol pathway, which covers for aldose reductase and polyol dehydrogenase activities, was determined in the testicular tissues using previously described protocols [40, 41].

#### 2.15.1 Aldose Reductase Activity

Briefly, 100 µL of the tissue supernatant was mixed with 700 µL of phosphate buffer (pH 6.7), 100 µL of 0.25 mM NADPH, and 100 µL of DL‐glyceraldehyde. Absorbance was read at 340 nm for 3 min at 30 s intervals using a microplate reader.

#### 2.15.2 Polyol Dehydrogenase Activity

Briefly, 10 µL of the tissue supernatant was mixed with 240 µL of 50 mM glycine/NaOH buffer (pH 10.0) containing 57 mM sorbitol in a 96‐well plate. Fifty microliter of 50 mM NAD^+^ was added to the reaction mixture. Absorbance was read at 340 nm at 30 s intervals for 3–5 min using a microplate reader.

### 2.16 Glucose 6‐Phosphate Dehydrogenase (G6PDH) Activity

The G6PDH activity of the testicular tissues was determined using a G6PDH ELISA kit (Merck, Johannesburg, South Africa; Catalog No: E‐BC‐K056‐M) according to the manufacturer's instructions.

### 2.17 Glyoxalase 1 Activity

The glyoxalase 1 (GLO1) activity of the testicular tissues was determined using a previously described protocol [42]. Briefly, 50 µL 2 mM methylglyoxal solution and 2 mM reduced glutathione (GSH) were incubated with an equal volume of 50 mM phosphate buffer (pH 6.6) at 37°C for 30 min. Ten microliter of the tissue supernatant was then added to the reaction mixture and further incubated for 10 min. Absorbance was read four times at 240 nm at 2‐min interval.

### 2.18 Glutathione Metabolism

Glutathione metabolism, which covers reduced glutathione (GSH) level, glutathione reductase, and glutathione peroxidase activities, was determined in the testicular tissues using previously described protocols [43, 44, 45].

#### 2.18.1 GSH Level

Briefly, 150 µL of the tissue supernatant was mixed with equal volume of 10% TCA and centrifuged at 2000 rpm for 10 min at room temperature. Eighty microliter of the supernatant was mixed with 40 µL of 0.5 mM DTNB in a 96‐well plate. The reaction mixture was further incubated with 200 µL of 0.2 M phosphate buffer (pH 7.8) for 15 min at room temperature. Absorbance was read at 415 nm. A GSH standard graph was used to extrapolate the GSH level.

#### 2.18.2 Glutathione Reductase Activity

Briefly, 10 µL of the tissue supernatant was mixed with 221 µL of 50 mM Tris–HCl buffer (containing 1 mM EDTA, pH 8.0) and 38 µL of 8 mM oxidized glutathione (GSSG) in a 96‐well plate. Ten microliter of NADPH was thereafter added to the reaction mixture. Absorbance was read at 340 nm at a 2‐min interval for 8 min using a microplate reader.

#### 2.18.3 Glutathione Peroxidase Activity

Briefly, 5 µL of the tissue supernatant was incubated with 210 µL of phosphate buffer (pH 6.9), 2.5 µL of 100 mM GSH, and 5 µL of distilled H_2_O in a 96‐well plate at 37°C for 10 min. About 2.5 µL of 15 mM NADPH was added to the reaction mixture, followed by 10 µL of 1 mM DTNB. Absorbance was read at 412 nm using a microplate reader. A GSH standard graph was used to extrapolate the glutathione peroxidase activity.

### 2.19 Nucleotide Metabolism

Nucleotide metabolism, which covers ATP level, E‐NTPDase, and ATPase activities, was determined in the testicular tissues using previously described protocols [46, 47].

#### 2.19.1 ATP Level

Briefly, 50 µL of the tissue supernatant was incubated with an equal volume of CellTiter‐Glo reagent in an opaque 96‐well plate on a shaker at 25°C in the dark for 30 min. Luminescence was measured using a microplate reader.

#### 2.19.2 E‐NTPDase Activity

Briefly, 20 µL of the tissue supernatant was incubated with 200 µL of a reaction buffer (1.5 mM CaCl_2_, 5 mM KCl, 0.1 mM EDTA, 10 mM glucose, 225 mM sucrose, and 45 mM Tris–HCl) for 10 min at 37°C. Twenty microliter of 50 mM ATP was thereafter added to the reaction mixture and further incubated in a shaker at 37°C for 20 min. The reaction was stopped with 200 µL of 10% TCA. Two hundred microliter of 1.25% ammonium molybdate and a freshly prepared 9% ascorbic acid were thereafter added to the reaction mixture. The reaction mixture was allowed to stand on ice for 10 min, and the absorbance was read at 600 nm.

#### 2.19.3 ATPase Activity

Briefly, 200 µL of the tissue supernatant was incubated with 200 µL of 5 mM KCl, 1300 µL of 0.1 M Tris–HCl buffer, and 40 µL of 50 mM ATP in a shaker at 37°C for 30 min. One microliter of distilled water and ammonium molybdate were added to the reaction mixture to terminate the reaction. Freshly prepared 9% ascorbic acid was added to the mixture and allowed to stand on ice for 10 min. Absorbance was measured at 660 nm.

### 2.20 Histology

Following fixation in 10% neutral buffered saline, the testicular tissues were routinely processed in paraffin. The processed tissues were further subjected to hematoxylin and eosin staining. Digital photomicrographs were obtained after viewing slides with a Digital Bright‐field Microscope (OMAX, USA) [48].

### 2.21 Statistical Analysis

Data were analyzed with One‐way analysis of variance (ANOVA) and presented as mean ± SD, with significant differences between means obtained at *p* < 0.05 compared to either NC or DBC. IBM Statistical Package for the Social Sciences (SPSS) for Windows, version23.0 (IBM Corp., Armonk, NY, USA) was used for the statistical analyses.

## 3 Results

Treatment of T2D rats with low and high doses of SPCs led to 65% and 67% decrease in blood glucose level, respectively.

**FIGURE 1:**
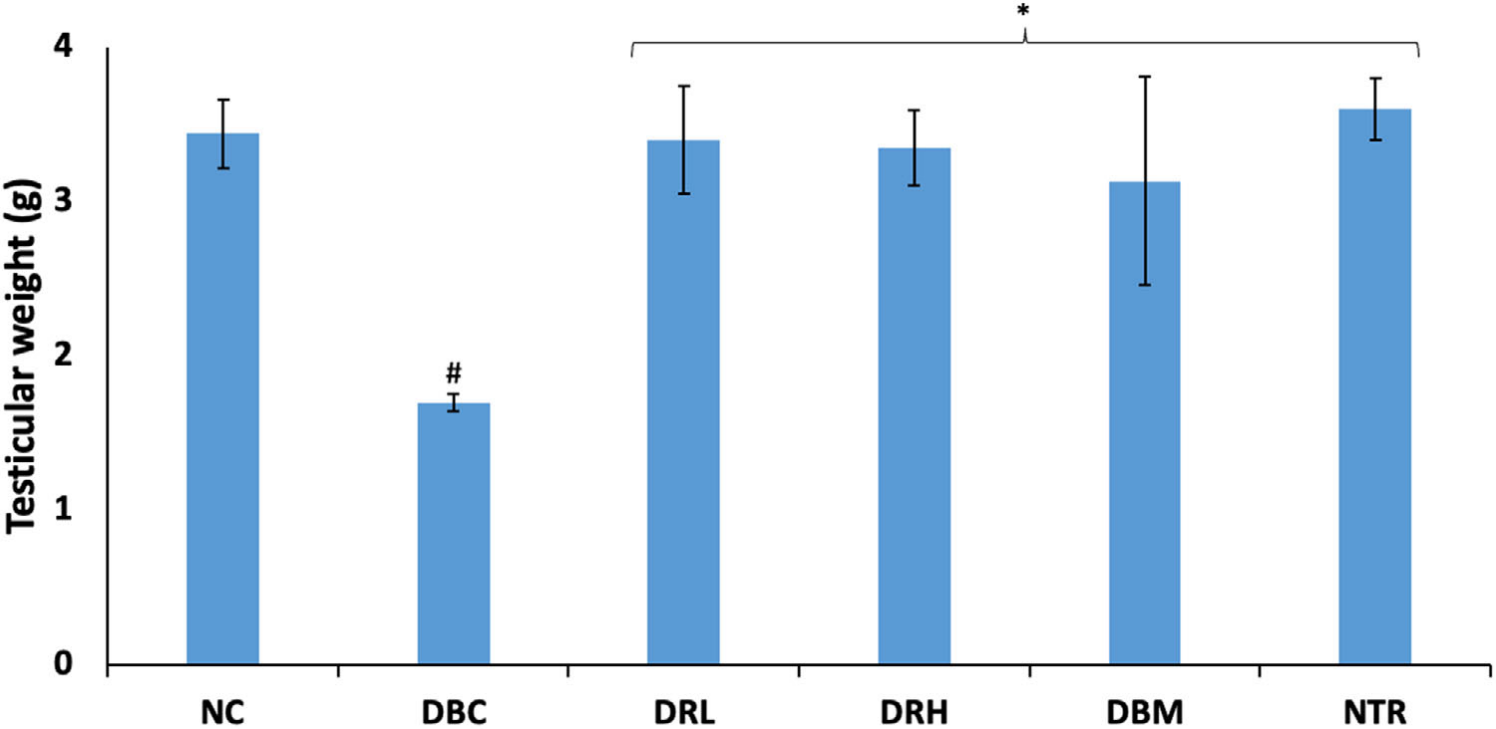
Effect of sulfated polysaccharides on testicular weights of diabetic rats. Statistical significance was determined by one‐way ANOVA. Statistical significance was evaluated with *p* < 0.05 compared to *DBC and #NC; *n* = 5 (NC and NTR) and 6 (DBC, DRL, DRH, and DBM). DBC indicates diabetic control; DBM, diabetic rats + metformin; DRH, diabetic rats + high dose SPCs; DRL, diabetic rats + low dose SPCs; NC, normal control; NTR, normal rats + high dose SPCs.

Induction of T2D significantly (*p* < 0.05) decreased the testicular weights of the rats, as shown in Figure 1. The weight was significantly (*p* < 0.05) increased following treatment with both doses of SPCs, and compared favorably with the standard drug (DBM) and normal control (NC) groups.

**FIGURE 2:**
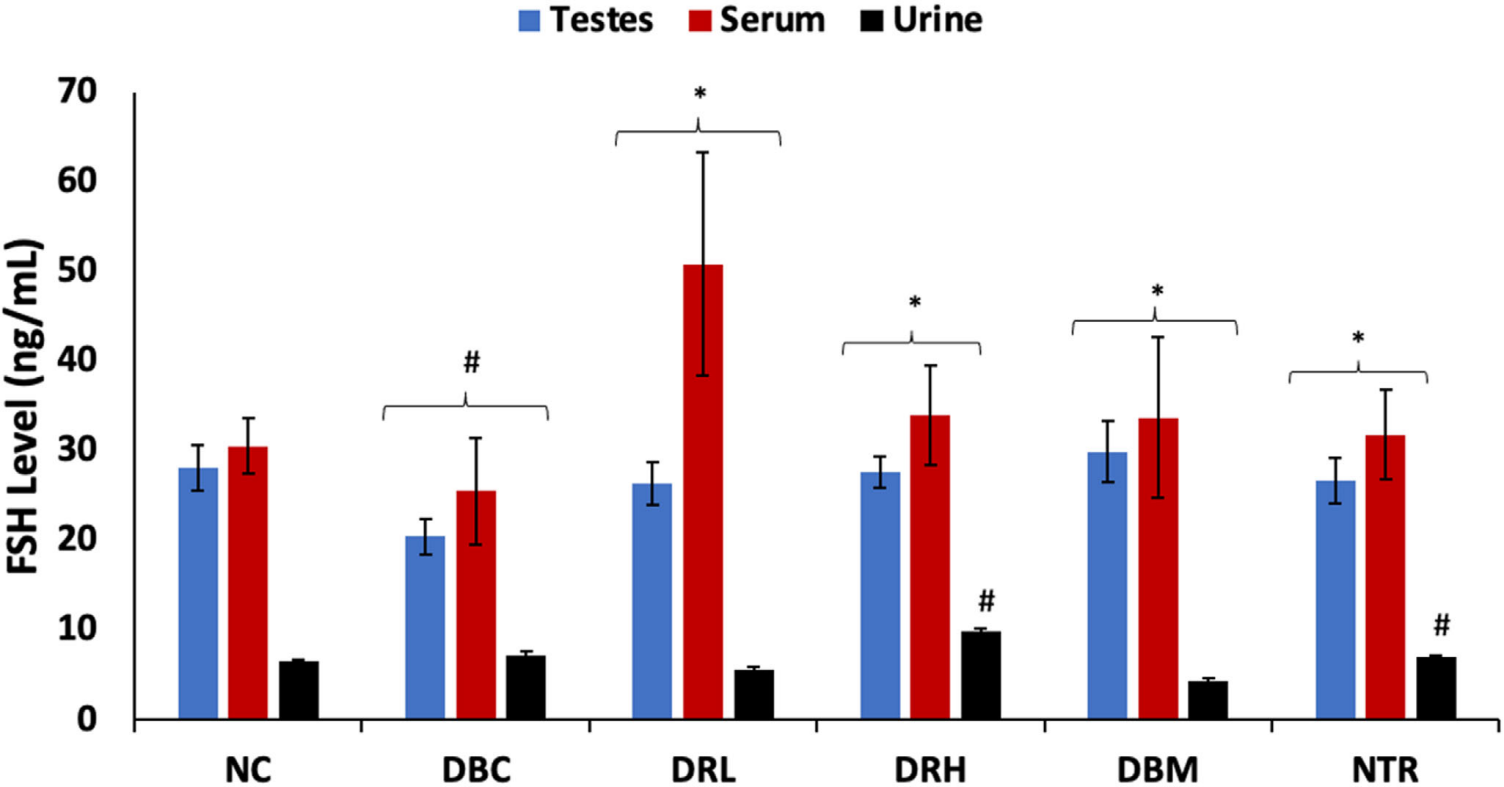
Effect of sulfated polysaccharides on FSH levels in testes, serum, and urine of diabetic rats. Statistical significance was evaluated with *p* < 0.05 compared to *DBC and #NC; *n* = 5 (NC and NTR) and 6 (DBC, DRL, DRH, and DBM). DBC indicates diabetic control; DBM, diabetic rats + metformin; DRH, diabetic rats + high dose SPCs; DRL, diabetic rats + low dose SPCs; NC, normal control; NTR, normal rats + high dose SPCs.

There was a significant (*p* < 0.05) decrease in serum and testicular levels of FSH following the induction of T2D, as shown in Figure 2. Induction of T2D also significantly (*p* < 0.05) elevated the urinal level of FSH. These levels were significantly (*p* < 0.05) reversed in serum and testicular tissues, following treatment with SPCs, with the low dose (150 mg/kg bw) having the best activity in the testes. Furthermore, a low dose of SPCs significantly (*p* < 0.05) decreased urinal levels of FSH.

**FIGURE 3:**
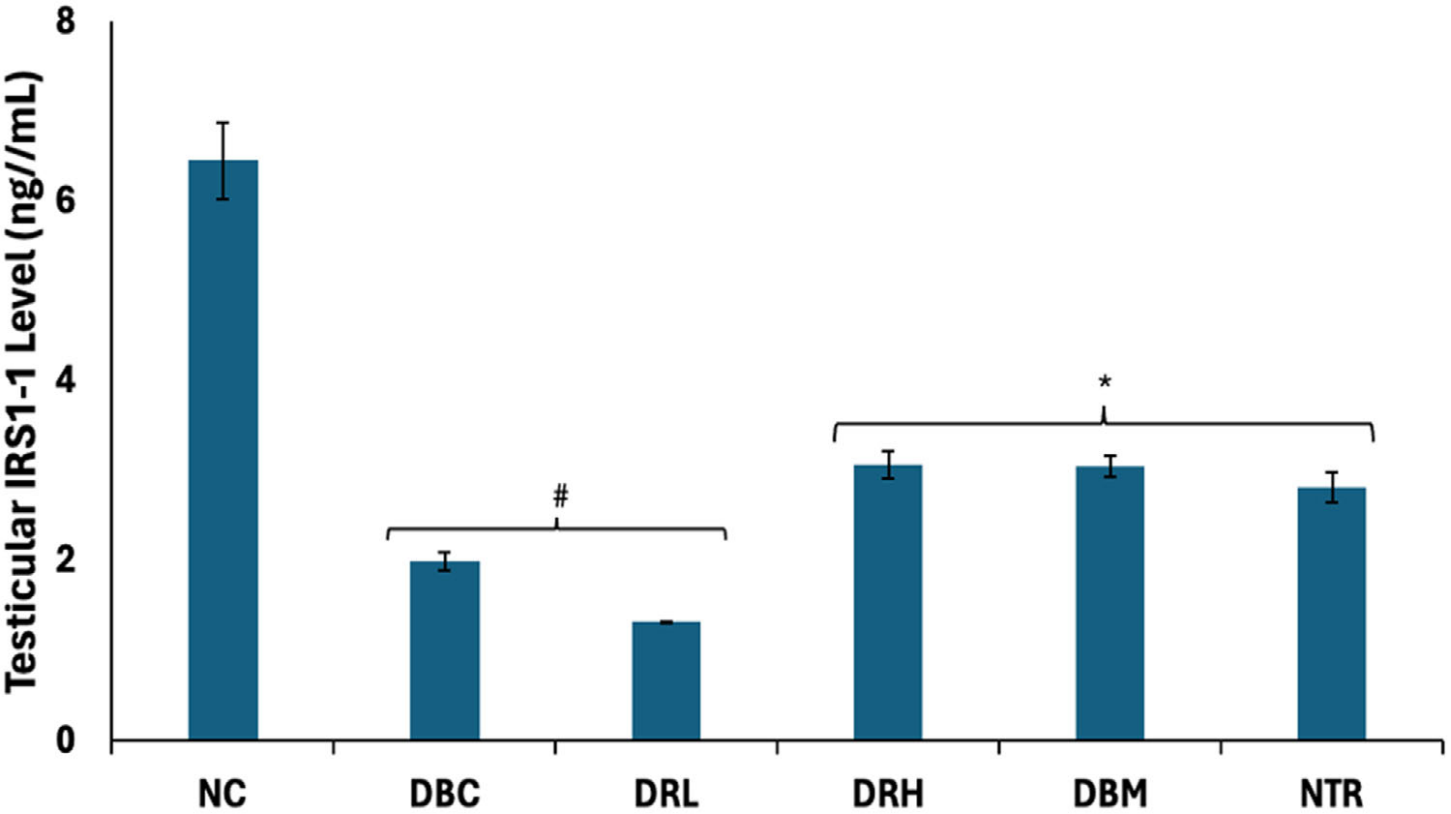
Effect of sulfated polysaccharides on testicular IRS‐1 level in diabetic rats. Statistical significance was determined by one‐way ANOVA. Statistical significance was evaluated with *p* < 0.05 compared to *DBC and #NC; *n* = 5 (NC and NTR) and 6 (DBC, DRL, DRH, and DBM). DBC indicates diabetic control; DBM, diabetic rats + metformin; DRH, diabetic rats + high dose SPCs; DRL, diabetic rats + low dose SPCs; NC, normal control; NTR, normal rats + high dose SPCs.

As shown in Figure 3, the testicular protein level of IRS‐1 was significantly (*p* < 0.05) decreased following induction of T2D. Except for the low dose group (DRL), the protein levels were significantly (*p* < 0.05) elevated following treatment with SPCs.

**FIGURE 4:**
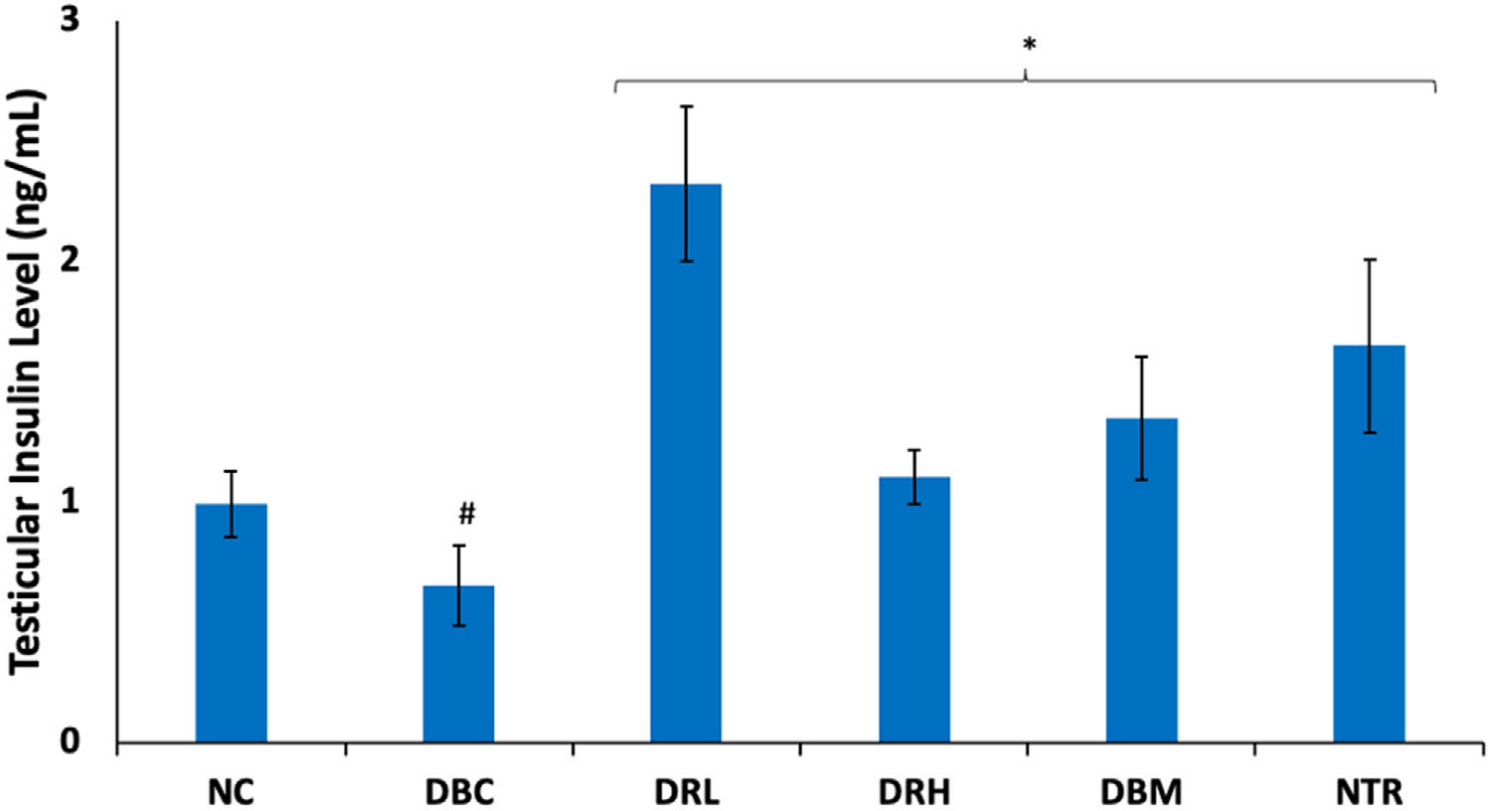
Effect of sulfated polysaccharides on testicular insulin level in diabetic rats. Statistical significance was determined by one‐way ANOVA. Statistical significance was evaluated with *p* < 0.05 compared to *DBC and #NC; *n* = 5 (NC and NTR) and 6 (DBC, DRL, DRH, and DBM). DBC indicates diabetic control; DBM, diabetic rats + metformin; DRH, diabetic rats + high dose SPCs; DRL, diabetic rats + low dose SPCs; NC, normal control; NTR, normal rats + high dose SPCs.

Induction of T2D led to a significant (*p* < 0.05) decrease in testicular insulin levels, as shown in Figure 4. These levels were significantly (*p* < 0.05) increased following treatment with SPCs, with the low dose having the best activity.

**FIGURE 5:**
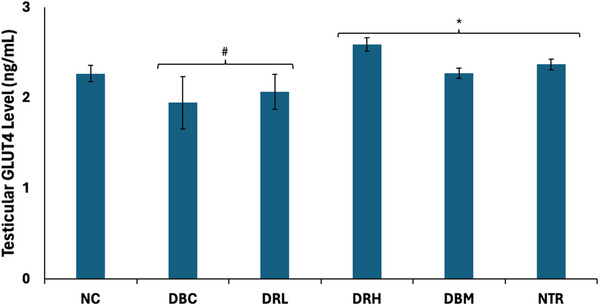
Effect of sulfated polysaccharides on testicular GLUT4 level in diabetic rats. Statistical significance was determined by one‐way ANOVA. Statistical significance was evaluated with *p* < 0.05 compared to *DBC and #NC; *n* = 5 (NC and NTR) and 6 (DBC, DRL, DRH, and DBM). DBC indicates diabetic control; DBM, diabetic rats + metformin; DRH, diabetic rats + high dose SPCs; DRL, diabetic rats + low dose SPCs; NC, normal control; NTR, normal rats + high dose SPCs.

The testicular GLUT4 levels were significantly (*p* < 0.05) depleted following induction of T2D, as shown in Figure 5. At high dose (300 mg/kg bw), treatment with SPCs significantly (*p* < 0.05) elevated the level.

**FIGURE 6:**
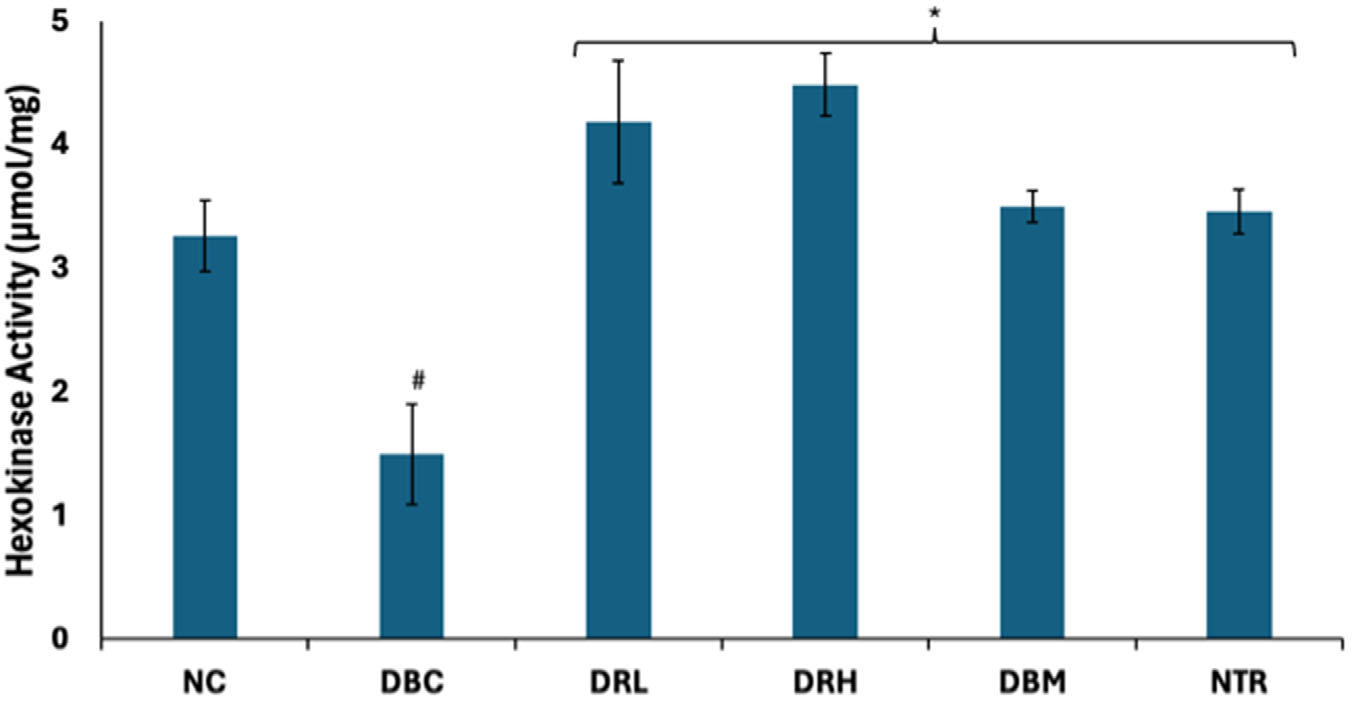
Effect of sulfated polysaccharides on hexokinase activity in testes of diabetic rats. Statistical significance was determined by one‐way ANOVA. Statistical significance was evaluated with *p* < 0.05 compared to *DBC and #NC; *n* = 5 (NC and NTR) and 6 (DBC, DRL, DRH, and DBM). DBC indicates diabetic control; DBM, diabetic rats + metformin; DRH, diabetic rats + high dose SPCs; DRL, diabetic rats + low dose SPCs; NC, normal control; NTR, normal rats + high dose SPCs.

As shown in Figure 6, the hexokinase activity of the testicular tissues was significantly (*p* < 0.05) suppressed following the induction of T2D. The activity was significantly (*p* < 0.05) elevated following treatment with SPCs.

**FIGURE 7:**
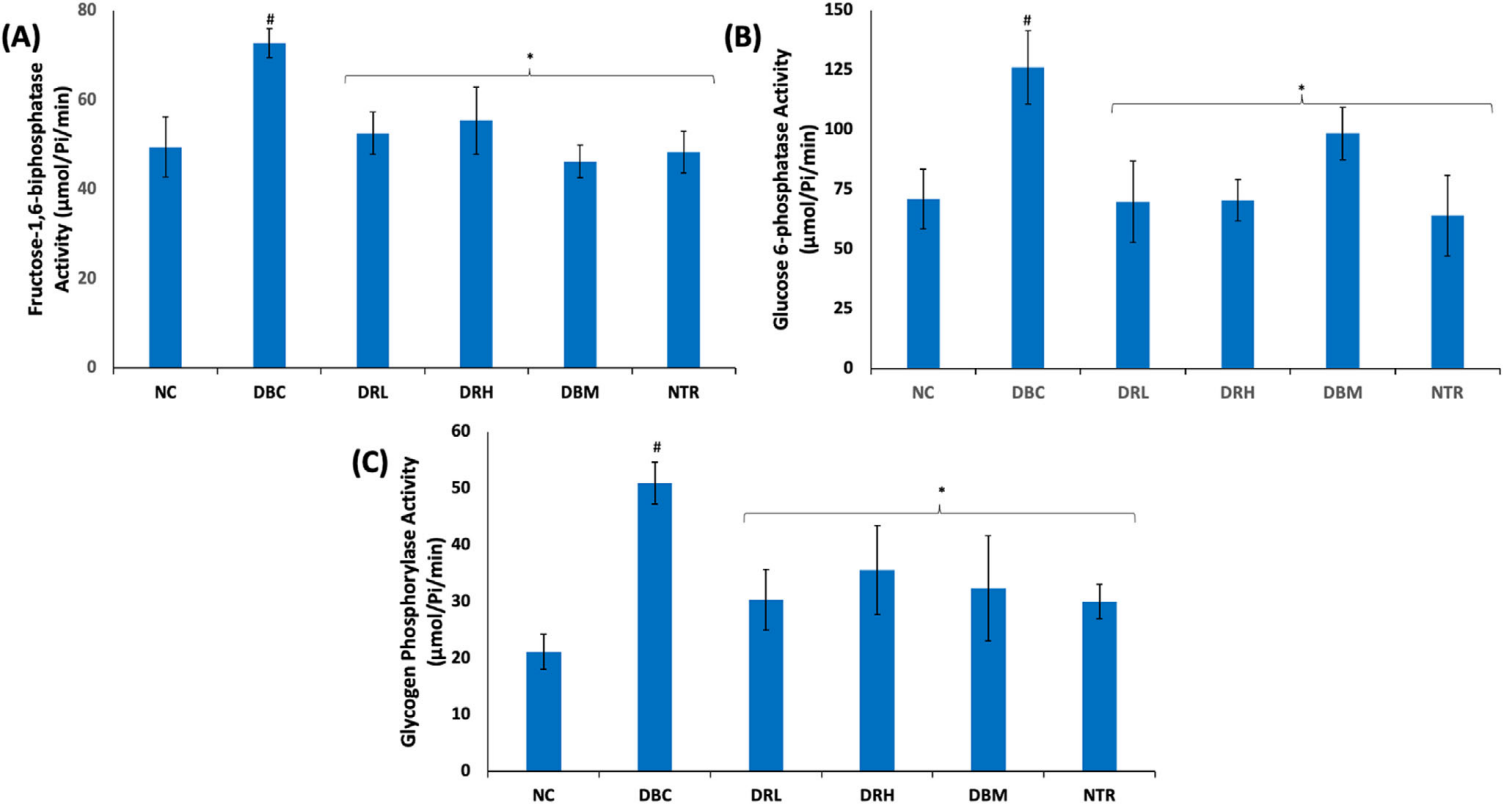
Effect of sulfated polysaccharides on glucogenic activities in testes of diabetic rats. (A) Fructose‐1,6‐biphosphatase; (B) glucose 6‐phosphatase; and (C) glycogen phosphorylase activities. Statistical significance was determined by one‐way ANOVA. Statistical significance was evaluated with *p* < 0.05 compared to *DBC and #NC; *n* = 5 (NC and NTR) and 6 (DBC, DRL, DRH, and DBM). DBC indicates diabetic control; DBM, diabetic rats + metformin; DRH, diabetic rats + high dose SPCs; DRL, diabetic rats + low dose SPCs; NC, normal control; NTR, normal rats + high dose SPCs.

As shown in Figure 7A–C, induction of T2D led to a significant (*p* < 0.05) increased in testicular glucogenic activities as depicted by elevated activities of fructose‐1,6‐biphosphatase, glucose 6‐phosphatase, and glycogen phosphorylase. These activities were significantly (*p* < 0.05) reversed following treatment with SPCs.

**FIGURE 8:**
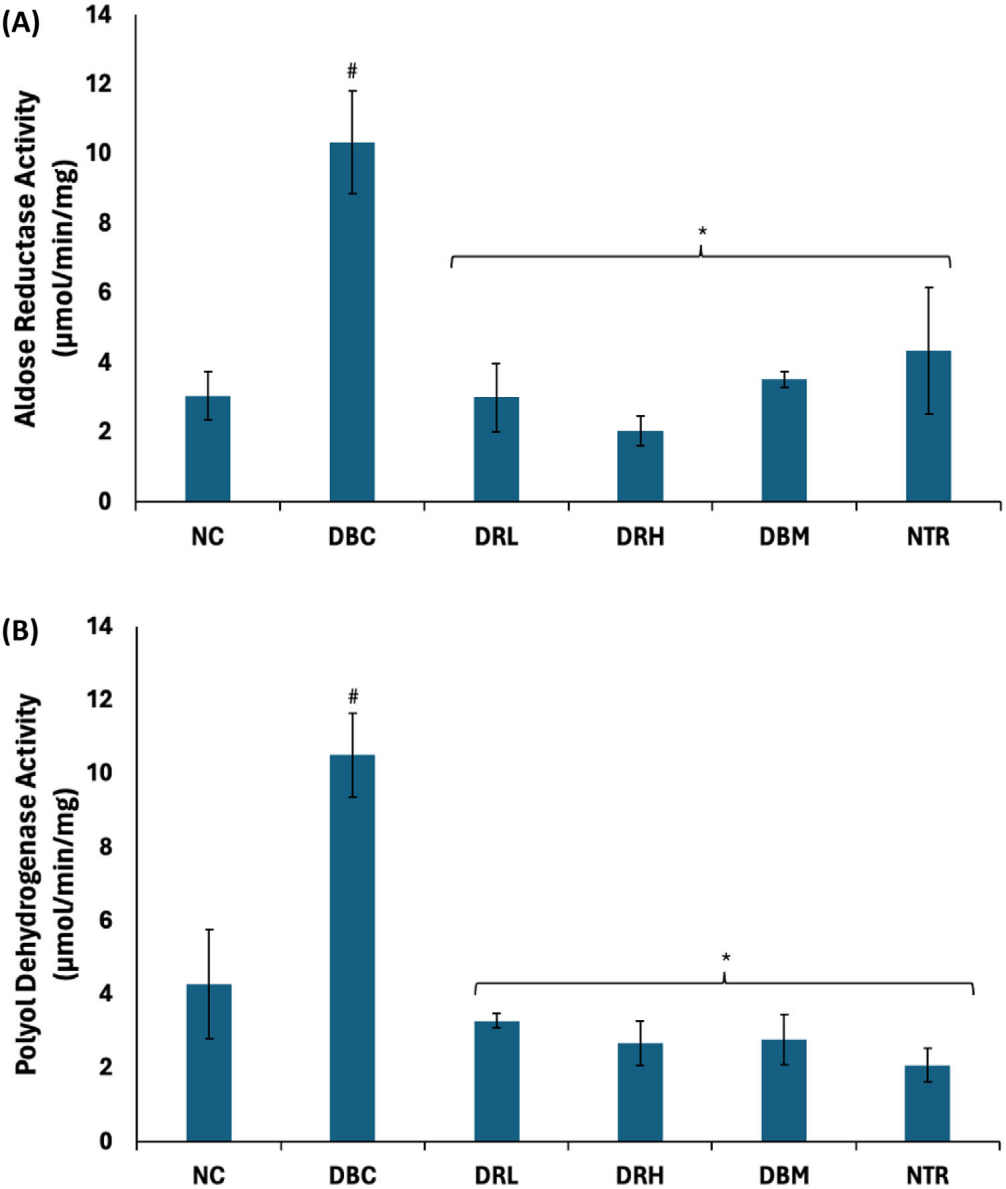
Effect of sulfated polysaccharides on polyol metabolism in testes of diabetic rats. (A) Aldose reductase and (B) polyol dehydrogenase activities. Statistical significance was determined by one‐way ANOVA. Statistical significance was evaluated with *p* < 0.05 compared to *DBC and #NC; *n* = 5 (NC and NTR) and 6 (DBC, DRL, DRH, and DBM). DBC indicates diabetic control; DBM, diabetic rats + metformin; DRH, diabetic rats + high dose SPCs; DRL, diabetic rats + low dose SPCs; NC, normal control; NTR, normal rats + high dose SPCs.

The testicular activities of aldose reductase and polyol dehydrogenase were significantly (*p* < 0.05) elevated following the induction of T2D as shown in Figure 8A,B. Treatment with SPCs significantly (*p* < 0.05) reversed these enzymes activities to near normal.

**FIGURE 9:**
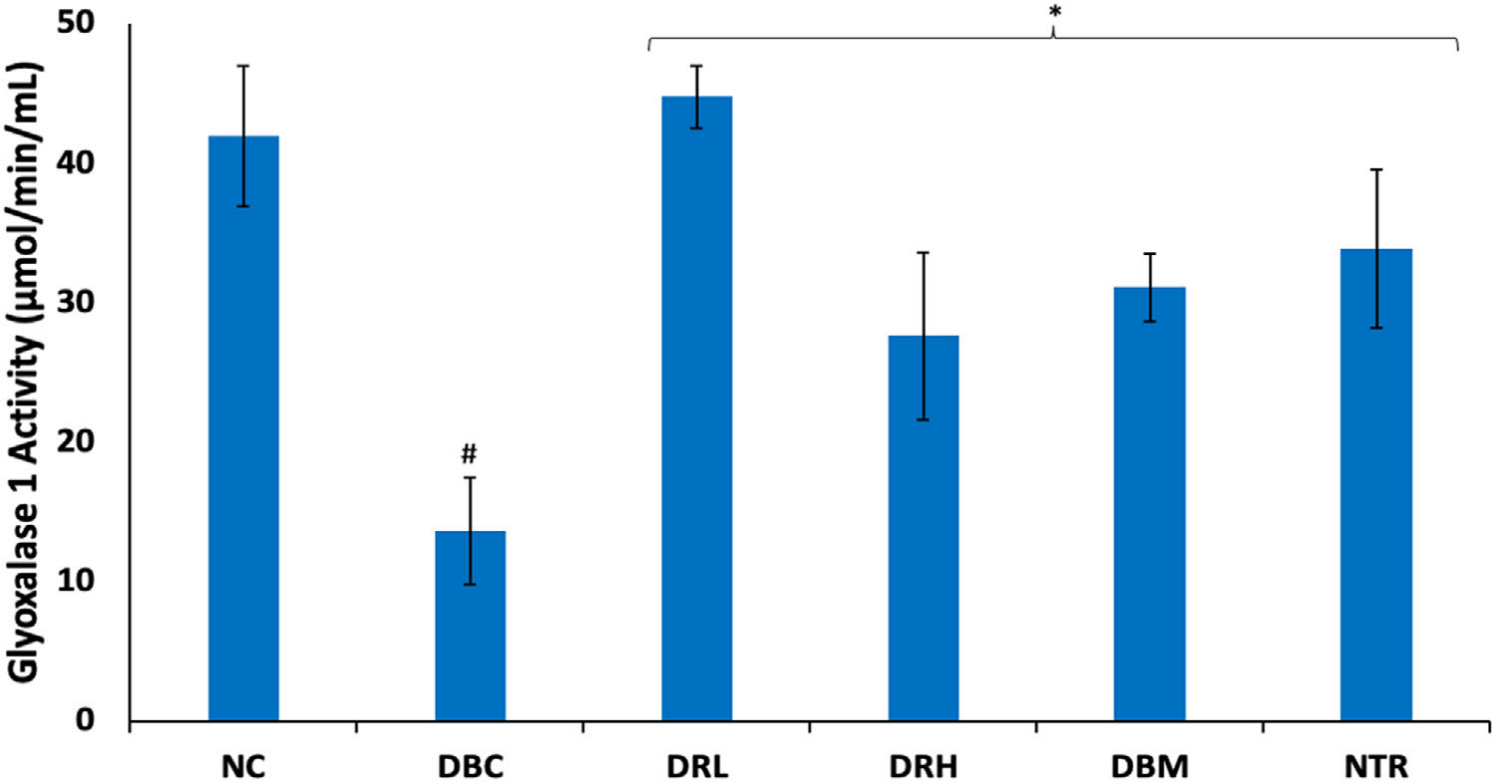
Effect of sulfated polysaccharides on GLO1 activity in testes of diabetic rats. Statistical significance was determined by one‐way ANOVA. Statistical significance was evaluated with *p* < 0.05 compared to *DBC and #NC; *n* = 5 (NC and NTR) and 6 (DBC, DRL, DRH, and DBM). DBC indicates diabetic control; DBM, diabetic rats + metformin; DRH, diabetic rats + high dose SPCs; DRL, diabetic rats + low dose SPCs; NC, normal control; NTR, normal rats + high dose SPCs.

As shown in Figure 9, induction of T2D led to significant (*p* < 0.05) depleted testicular activity of GLO1. Treatment with SPCs significantly (*p* < 0.05) elevated the activity, with the low dose depicting the best activity.

**FIGURE 10:**
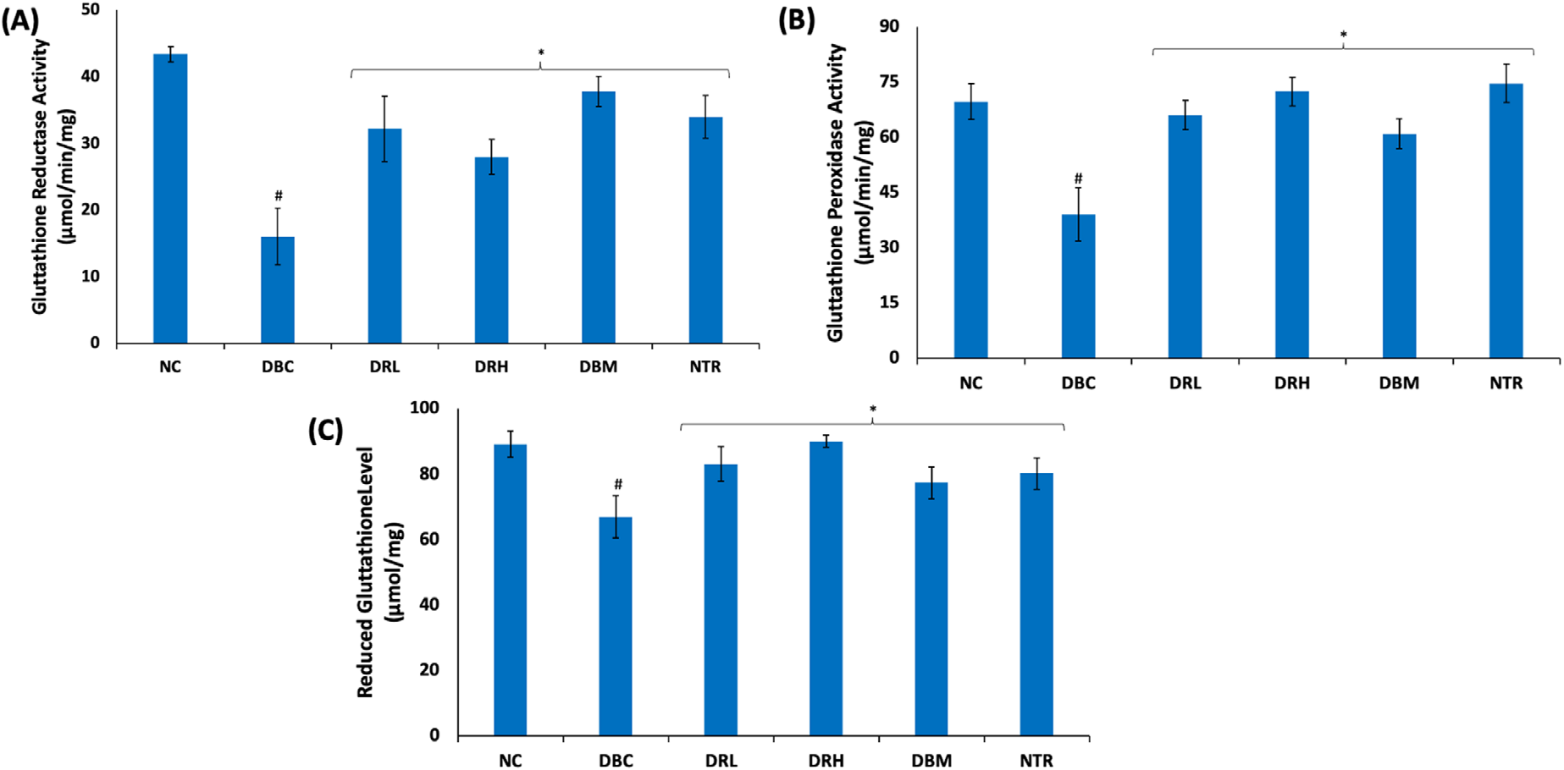
Effect of sulfated polysaccharides on glutathione metabolism in testes of diabetic rats. (A) Glutathione reductase; (B) glutathione peroxidase activities; and (C) reduced GSH level. Statistical significance was determined by one‐way ANOVA. Statistical significance was evaluated with *p* < 0.05 compared to *DBC and #NC; *n* = 5 (NC and NTR) and 6 (DBC, DRL, DRH, and DBM). DBC indicates diabetic control; DBM, diabetic rats + metformin; DRH, diabetic rats + high dose SPCs; DRL, diabetic rats + low dose SPCs; NC, normal control; NTR, normal rats + high dose SPCs.

There was a significant (*p* < 0.05) depletion in testicular activities of glutathione reductase and glutathione peroxidase activities, and GSH level, as shown in Figure 10A–C. These activities and levels were significantly (*p* < 0.05) reversed following treatment with SPCs.

**FIGURE 11:**
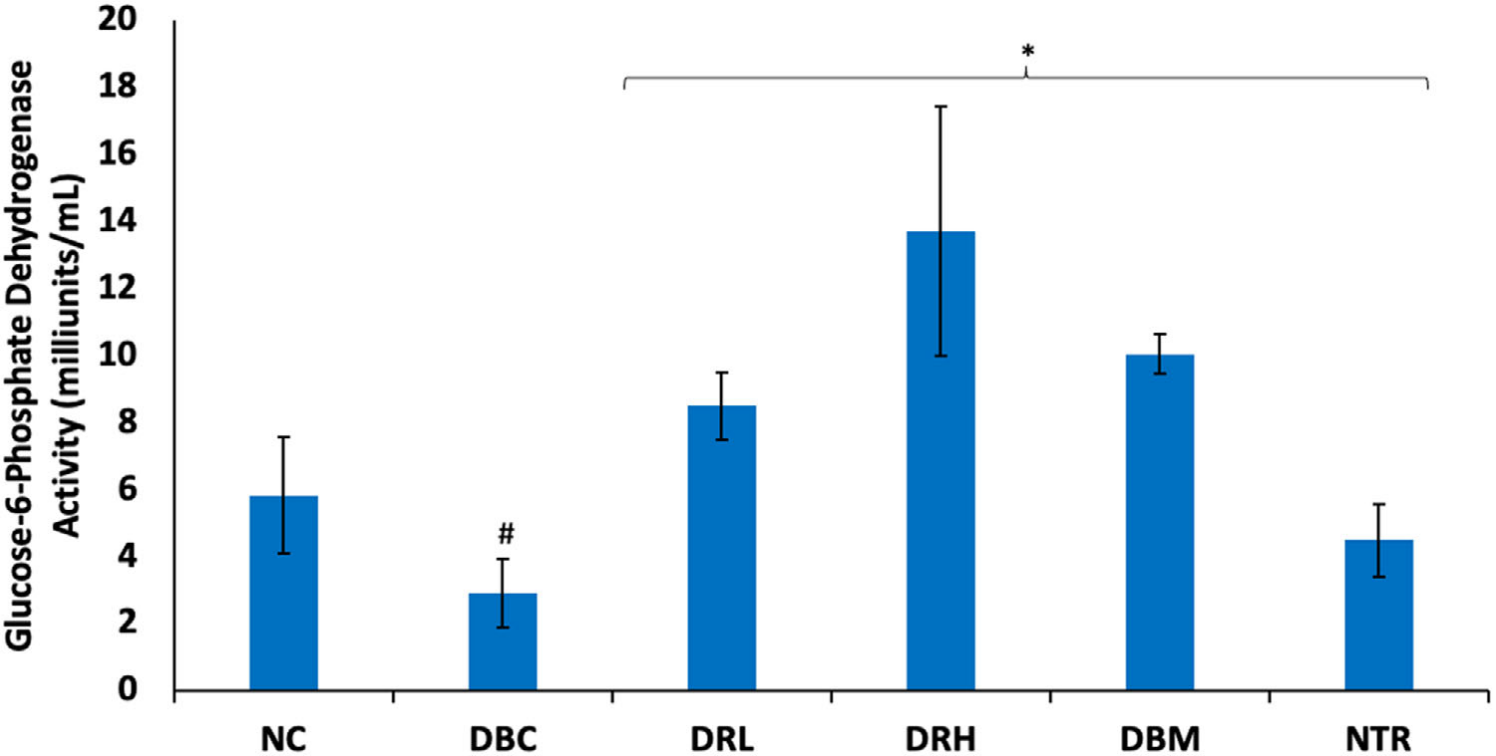
Effect of sulfated polysaccharides on glucose 6‐phosphate dehydrogenase activity in testes of diabetic rats. Statistical significance was determined by one‐way ANOVA. Statistical significance was evaluated with *p* < 0.05 compared to *DBC and #NC; *n* = 5 (NC and NTR) and 6 (DBC, DRL, DRH, and DBM). DBC indicates diabetic control; DBM, diabetic rats + metformin; DRH, diabetic rats + high dose SPCs; DRL, diabetic rats + low dose SPCs; NC, normal control; NTR, normal rats + high dose SPCs.

There was a significant (*p* < 0.05) depletion in testicular glucose 6‐phosphate dehydrogenase activity following the induction of T2D, as shown in Figure 11. The activity was significantly (*p* < 0.05) reversed following treatment with SPCs, with the high dose depicting the best activity.

**FIGURE 12:**
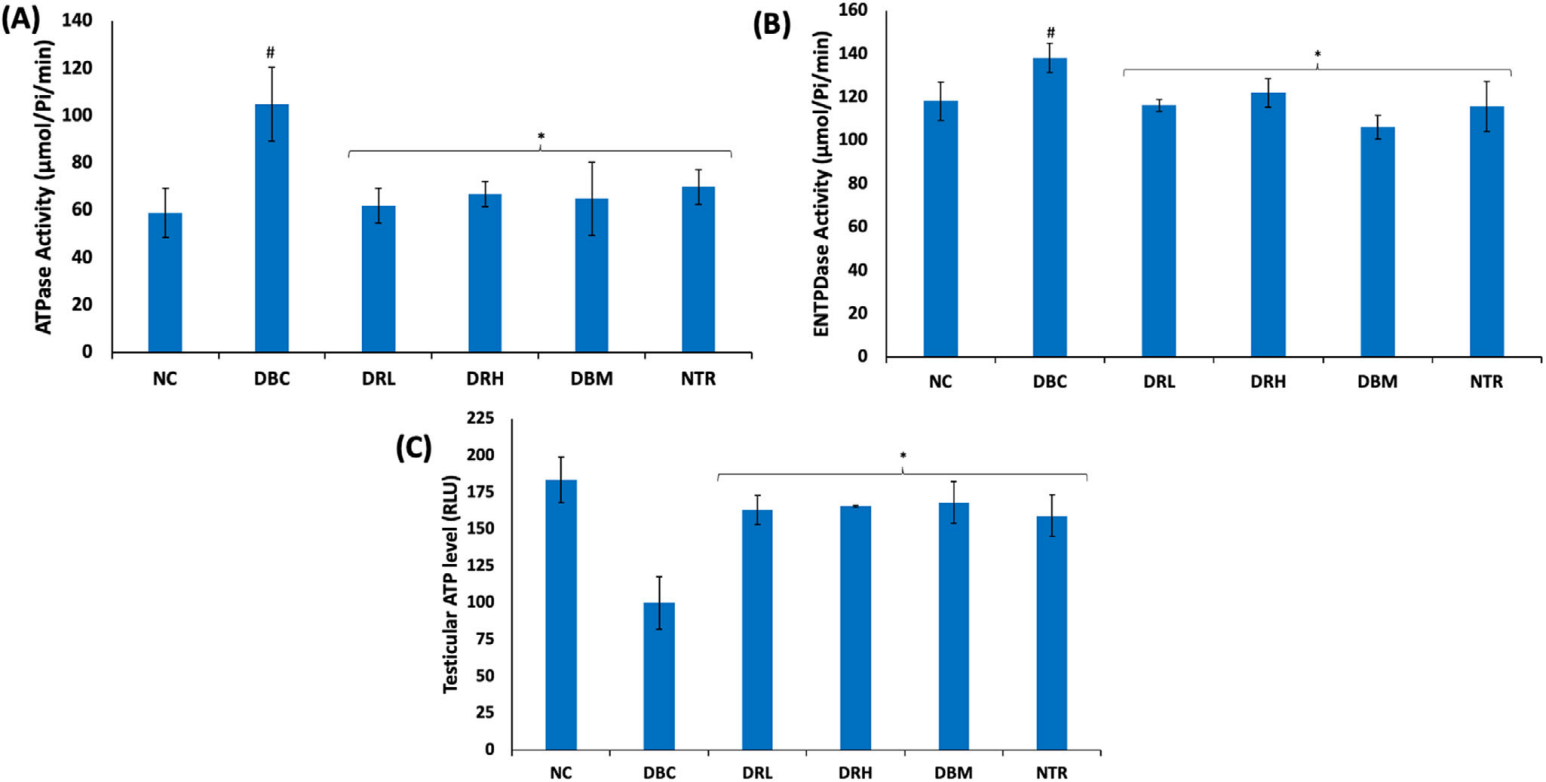
Effect of sulfated polysaccharides on nucleotide metabolism in testes of diabetic rats. (A) ATPase; (B) ENTPDase activities; (C) ATP level. Statistical significance was determined by one‐way ANOVA. Statistical significance was evaluated with *p* < 0.05 compared to *DBC and #NC; *n* = 5 (NC and NTR) and 6 (DBC, DRL, DRH, and DBM). DBC indicates diabetic control; DBM, diabetic rats + metformin; DRH, diabetic rats + high dose SPCs; DRL, diabetic rats + low dose SPCs; NC, normal control; NTR, normal rats + high dose SPCs.

Induction of T2D led to significant (*p* < 0.05) elevation in the testicular activities of ATPase and ENTPDase, while concomitantly suppressing ATP level as shown in Figure 12A–C. These activities and levels were significantly (*p* < 0.05) reversed following treatment with SPCs.

**FIGURE 13:**
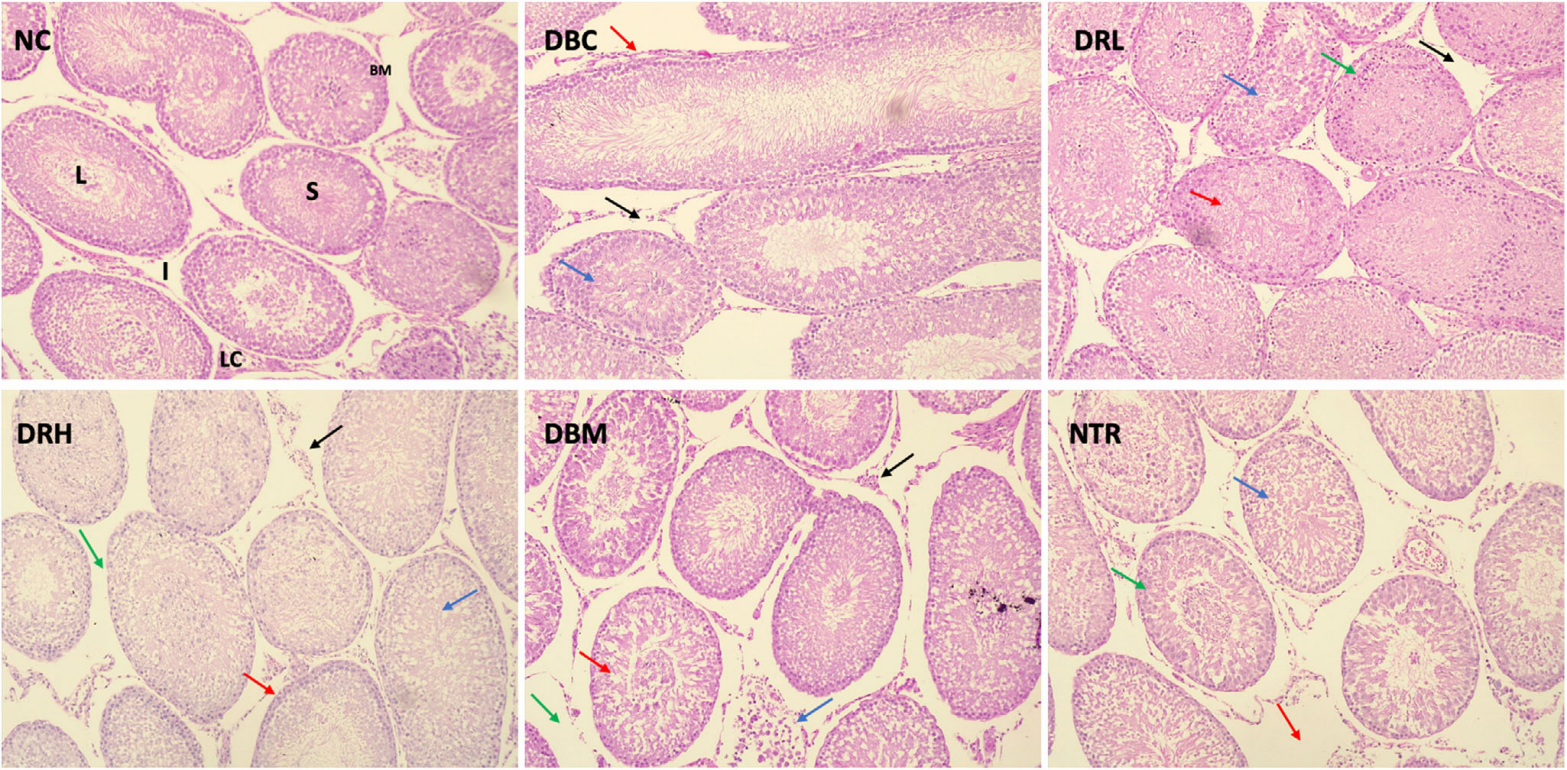
Effect of sulfated polysaccharides on testicular histology (H&E staining) of diabetic rats. Magnification = 40x. DBC indicates diabetic control; DBM, diabetic rats + metformin; DRH, diabetic rats + high dose SPCs; DRL, diabetic rats + low dose SPCs; NC, normal control; NTR, normal rats + high dose SPCs. **Legends: NC** = **BM**: basement membrane, **I**: interstitial spaces, **LC**: Leydig cells, **L**: lumen, and **S**: sperm; **DBC** = **red arrow**: detached basement membrane, **blue arrow**: congested seminiferous tubule, and **brown arrow**: degenerated Leydig cells; **DRL** = **blue arrow**: congested seminiferous tubule, **green arrow**: degenerated spermatozoa, **red arrow**: disarranged spermatogonia, and **black arrow**: narrow interstitial spaces; **DRH** = brown arrow: regenerated Leydig cells, **red arrow**: populated spermatogonia, **blue arrow**: less congested seminiferous tubules, and **black arrow**: narrow interstitial space. **DBM** = **brown arrow**: regenerated Leydig cells, **blue arrow**: less congested seminiferous tubules, **blue arrow**: degenerated basement membrane and seminiferous tubule, and **black arrow**: expanded interstitial space; and **NTR** = **green arrow**: expanded interstitial, **red arrow**: alteration of the basement membrane, **blue arrow**: congested seminiferous tubule.

As depicted in Figure 13, H&E staining revealed a well‐preserved cellular orientation in the basement membrane (BM), normal interstitial spaces (I) with well‐populated Leydig cells (LC), and normal lumen (L) with sperm cells in the lumen (S) of testicular tissues of normal control (NC). Induction of T2D led to the detachment of the basement membrane (red arrow), congestion of the seminiferous tubule (blue arrow), and degeneration of the Leydig cells (black arrow) as depicted in the DBC group. SPCs at low dose (DRL group) did not fully restore the testicular morphology as depicted by congested seminiferous tubule (red arrow), degenerated spermatozoa within the tubule (blue arrow), disarrangement of the spermatogonia (green arrow), and narrow interstitial spaces (black arrow). At high dose, SPCs led to regeneration of Leydig cells (black arrow), improved cellular orientation in the membrane basement, which is well populated with spermatogonia (red arrow), less congested seminiferous tubules (blue arrow), and a narrow interstitial space (green arrow) as depicted in the DRH group. Metformin led to regeneration of Leydig cells (black arrow), less congested seminiferous tubules (red arrow), degeneration of the membrane basement and seminiferous tubule (blue arrow), and expansion of the interstitial space (green arrow) as depicted in the DBM group. Furthermore, at high dose, SPCs led to widened interstitium or expansion of interstitial space (red arrow), alteration of the basement membrane (green arrow), and congestion of the seminiferous tubule (blue arrow) in testicular tissues of normal rats (NTR group).

## 4 Discussion

Testicular dysfunction arising from perturbed glucose metabolism, and characterized by impaired FSH and insulin signaling, is a major contributor to male infertility in T2D [15, 49]. The continuous search for an affordable and readily available treatment has led to a paradigm shift to natural products, with SPCs gaining much interest. In the present study, we investigated the effect of SPCs on glucose metabolism as well as FSH and insulin signaling in the testes of T2D rats.

Testicular atrophy has been linked as a contributory factor to male infertility in T2D [50, 51]. It is characterized by shrinkage of the testicles and reduced testicular weights, leading to morphological alterations that affect spermatogenesis and other testicular functions [52, 50]. These were corroborated in the present study by reduced testicular weights (Figure 1) and altered morphologies (Figure 13) in the untreated diabetic rats (DBC group). This also corroborates previous studies reporting altered testicular weights and morphology in diabetes [52, 51]. The seminiferous tubules are the sites of spermatogenesis, and their congestion has been implicated in depleted germ cells, leading to total or subtotal blockage of spermatogenesis [13, 53]. Degeneration of the Leydig cells causes a decrease in the production of testosterone and has been implicated in spermatogenesis disruption and decreased sperm quality [54]. The improved testicular weight and morphology following treatment with SPCs, particularly at the high dose as depicted in the DRH group, indicate restorative effects. These improvements suggest improved spermatogenesis and correspond with previous studies on the restorative effects of SPCs on testicular weight and morphology [29].

FSH plays an important role in male fertility but its activity is dependent on insulin/IGF‐1 signaling [15, 55]. Thus, indicating a synergistic effect between FSH and insulin in promoting Sertoli cell proliferation and spermatogenesis [56, 15]. However, their secretions and signaling are altered in diabetes [13, 57, 58]. The binding of FSH to its receptor in the testis causes protein kinase A (PKA)‐dependent phosphorylation of IRS‐1, which increases the sensitivity of IRS‐1 to insulin/IGF‐1 bound‐receptors [15]. The phosphorylation and activation of IRS‐1 by insulin/IGF‐1 bound‐receptors then triggers the PI3K‐AKT pathway, which drives the reaction cascade that stimulates Sertoli cell proliferation and spermatogenesis [15, 57, 59]. Thus, indicating IRS‐1 as the connecting hub for FSH and insulin synergistic activities in testicular functions and male reproduction. The depleted serum and testicular levels of FSH in untreated T2D rats (Figure 2) suggest impaired secretion of the hormone as reported in diabetics [58]. Furthermore, the elevated urinal level of FSH suggests gonadal disorder and testicular dysfunctions, which have also been reported in T2D [60]. The suppressed testicular levels of IRS‐1 (Figure 3) in the untreated T2D rats indicate an alteration in FSH and insulin signaling. This is further corroborated by the depleted testicular level of insulin in the untreated T2D rats (Figure 4), which also suggests the inability of plasma insulin to cross the BTB, leading to its insufficiency for testicular functions. The reversed levels of these hormones and IRS‐1, in the SPCs‐treated T2D rats, indicate improved secretions and signaling. Thus, suggesting improved spermatogenic activities.

Activation of the PI3K‐AKT pathway following insulin phosphorylation of IRS‐1 triggers a series of signaling cascades, including phosphorylation of downstream substrates that stimulate GLUT4 translocation [61]. Thereby, promoting glucose uptake, which is then channeled into the glycolytic pathway, where it is phosphorylated by hexokinase to glucose‐6‐phosphate. Spermatogenesis is dependent on anaerobic glycolysis for the generation of ATP. However, GLUT4 translocation and glycolysis are altered in diabetes [4]. The depleted testicular GLUT4 level and hexokinase activity in the untreated T2D rats (Figures 5 and 6) depict impaired glucose uptake and glycolysis. This is further corroborated by the elevated activities of fructose‐1,6‐biphosphatase, glucose 6‐phosphatase, and glycogen phosphorylase (Figure 7A–C), which depict gluconeogenesis and glycogenolysis. This glycolytic‐glucogenic switch, in favor of the latter, is activated following downregulation of GLUT4, to compensate for depleted cellular glucose levels and impaired glucose uptake [49, 62]. The elevated GLUT4 level and reversed enzyme activities in the SPCs‐treated T2D rats indicate improved testicular glucose uptake, activation of glycolysis, and arrest of gluconeogenesis and glycogenolysis. Thus, depicting a glycolytic‐glucogenic switch in favor of the former. This corroborates previous reports on the ability of SPCs to upregulate GLUT4 and promote glucose uptake [27, 63]. However, these effects have not been reported in testicular tissues.

Incessant glucogenesis and glycogenolysis will lead to continuous generation of glucose, thereby exacerbating cellular glucose levels [4, 49]. However, these exacerbated glucose levels cannot be utilized by the cells for ATP production in diabetic states owing to impaired insulin signaling [64]. Thus, leading to their channeling to other pathogenic pathways such as the polyol, protein kinase C, AGE, and hexosamine pathways. These pathways generate toxic metabolites, which have been implicated in spermatogenic disruption [65, 66]. The elevated activities of aldose reductase and polyol dehydrogenase in the untreated diabetic rats (Figure 8A,B) suggest a channeling of excess cellular glucose to the polyol pathway. This pathway has been implicated in alteration of spermatogenesis, oxidative damage of sperm DNA, impaired sperm motility, and quality [66, 67]. It consists of two steps, where glucose is first converted to sorbitol by aldose reductase. Polyol dehydrogenase then converts sorbitol to fructose. The reversed activities of these enzymes following treatment with SPCs indicate an inhibition of the polyol pathway. Thus, suggesting the potential of the polysaccharides to suppress the production of fructose.

The produced fructose from incessant activation of polyol pathway has been implicated in the production of advanced glycation end products (AGEs) which contributes to spermatogenic disruption [65]. Fructose is phosphorylated and hydrolyzed to dihydroxyacetone phosphate (DHAP), which is further converted to methylglyoxal, a toxic metabolite [4, 68]. Methylglyoxal has been implicated in testicular atrophy, impaired spermatogenesis, sperm DNA fragmentation, and impaired sperm motility in diabetic states [68, 69, 70]. The detoxification of methylglyoxal involves the glyoxalase system, where it is first converted to S‐D‐lactoylglutathione by glyoxalase 1 (GLO1), and the latter is then converted to lactate by glyoxalase 2 (GLO2) [71]. Therefore, the decreased testicular GLO1 activity in the untreated T2D rats (Figure 9) indicates elevated cellular levels of methylglyoxal and correlates with previous reports on high levels in diabetes states [66, 70]. The exacerbated activity of the enzyme following treatment with SPCs indicates depleted cellular levels of methylglyoxal. Thus, indicating the ability of the sulfated polysaccharides to activate the glyoxylate system and mitigate methylglyoxal toxicity.

Reduced glutathione (GSH) is a cofactor for GLO1, and thus, its depletion alters the glyoxalase system [72]. Cellular levels of GSH are maintained by a balance between the activities of glutathione reductase and glutathione peroxidase. Glutathione reductase reduces oxidized glutathione (GSSG) to GSH, while glutathione peroxidase oxidizes GSH to GSSG. The decreased testicular activities of these enzymes in the untreated T2D rats (Figure 9A,B), corroborate the depleted GSH level (Figure 9C). Thus, indicating alteration in the function of the glyoxalase system and correlates with the suppressed activity of GLO1. The reversed enzyme activities and GSH level in SPCs‐treated rats, therefore, indicate a restorative effect on glutathione metabolism. This correlates with the improved GLO1 activity, and thus, an effective glyoxalase system. The restorative effect corroborates previous studies on the ability of sulfated polysaccharides to improve GSH level and the activities of glutathione reductase and glutathione peroxidase [73, 74].

Glucose 6‐phosphate from the glycolytic pathway can also be channeled to the pentose phosphate pathway (PPP) to generate NADPH, an important cofactor for various cellular activities, including spermatogenesis and glutathione reductase [75]. Glucose‐6‐phosphate dehydrogenase catalyzes the first step in the pathway, and its inhibition in diabetic testes has been linked to impaired spermatogenesis, sperm motility, and redox imbalance [76, 77]. Thus, the suppressed testicular G6PDH activity in the untreated T2D rats (Figure 11) indicates an inhibition of the enzyme activity and inactivation of the PP pathway. The elevated activity following treatment with SPCs, therefore, indicates activation of the PP pathway and improved cellular level of NADPH.

Diabetes alters testicular nucleotide metabolism which disrupts energy balance, leading to impaired spermatogenesis, sperm—motility, counts, and quality [78, 79]. It is characterized by suppressed ATP levels arising from arrested glycolytic phosphorylation and exacerbated activities of purinergic enzymes [4, 49]. The elevated testicular activities of the purinergic enzymes, ATPase, and ENTPDase in the untreated T2D rats (Figure 12A,B) indicate altered purinergic activities and distorted energy homeostasis [49]. This is further corroborated by the depleted ATP level (Figure 12C). These alterations may suggest suppressed spermatogenic activities and can be attributed to the glycolytic‐gluconeogenic switch in favor of the former. The reversed purinergic activities and elevated ATP level in SPCs‐treated rats depict improved energy homeostasis and correlates with the glycolytic‐gluconeogenic switch in favor of the latter.

**FIGURE 14:**
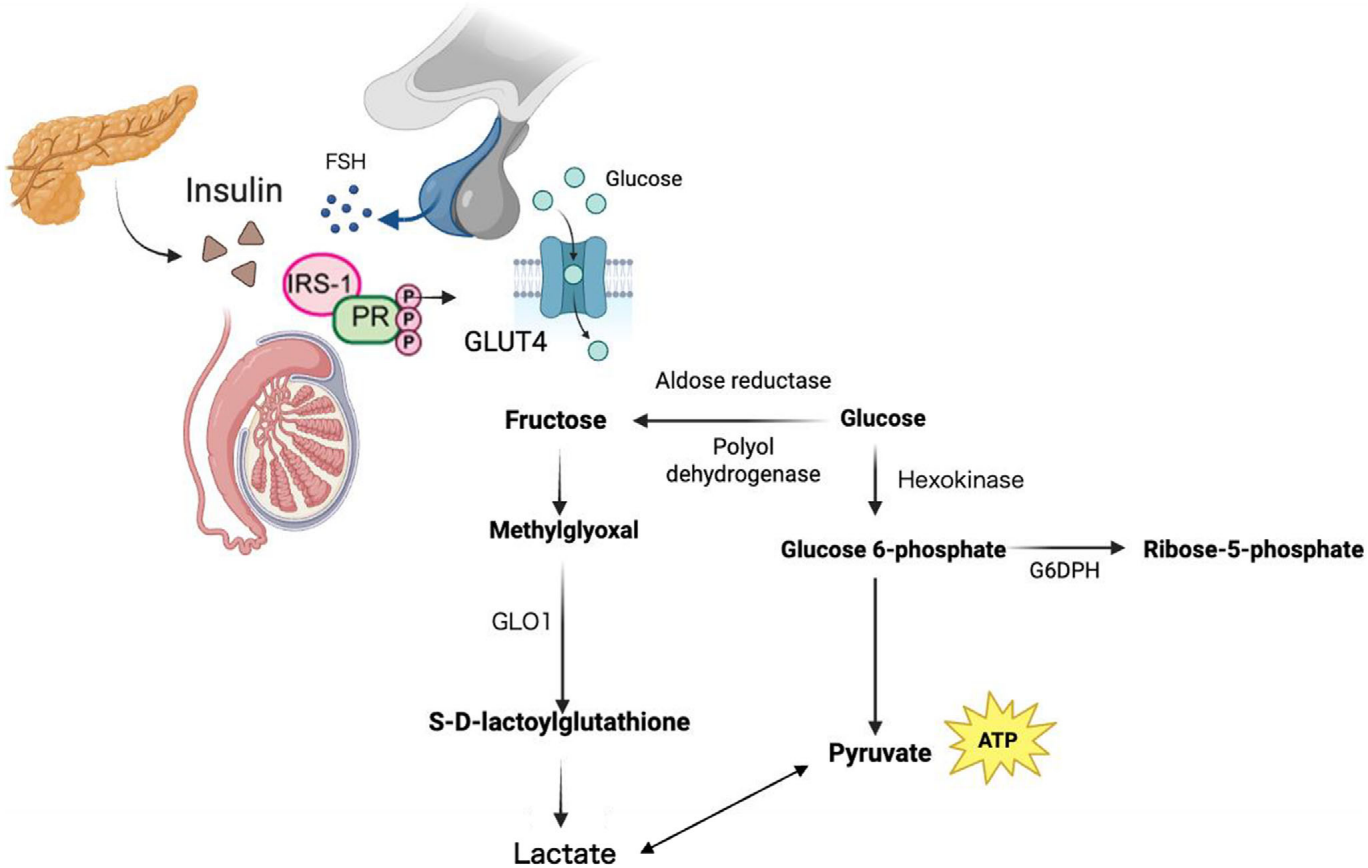
Schematic pathway summarizing the effect of SPCs on T2D‐mediated testicular dysfunction.

A schematic pathway summarizing the effect of SPCs on T2D mediated testicular dysfunction is shown in Figure 14.

The reduced testicular levels of IRS‐1 and glucose 6‐phosphate dehydrogenase activity in normal rats administered high dose of SPCs (Figures 3 and 11) indicate impaired insulin‐FSH signaling and inactivation of the PP pathway. This may be a cause of concern on the safety of SPCs on testicular activities of healthy males. However, this requires further studies as the present study lacks long‐term safety data.

## 5 Conclusion

The study indicates the protective effect of sulfated polysaccharides from *G. gracilis* on hyperglycemia‐mediated testicular dysfunction in T2D. It provides evidence that SPCs improves testicular glucose metabolism by stimulating FSH‐insulin signaling via the IRS‐1 hub. This is further portrayed by elevated GLUT4 level, improved energy homeostasis, and modulation of the glycolytic‐gluconeogenic metabolic switch. Furthermore, SPCs mitigated glucotoxicity via inhibiting the polyol pathway, while improving the glyoxalase system and glutathione metabolism. They also improved testicular atrophy and morphology. However, the study lacks data on sperm quality and long‐term safety of SPCs. These limitations require further investigations to fully decipher the protective effect of SPCs in managing T2D testicular dysfunction. Furthermore, the translational relevance of SPCs in clinical studies may warrant trials of doses lower than 300 mg/kg bw.

## Ethics Statement

This study protocol was approved by the Animal Research Ethics Committee of the University of KwaZulu‐Natal, Durban, South Africa (Ethical Approval Number: AREC/00002347/2021).

## Conflicts of Interest

The authors declare no conflicts of interest.

## Data Availability

The data in support of this research findings will be made available on reasonable request.
